# Case Report: Priapism as the clinical presentation of chronic myeloid leukemia in accordance with reports created during last twenty years: a case report and literature review

**DOI:** 10.12688/f1000research.53365.1

**Published:** 2021-07-15

**Authors:** Siprianus Ugroseno Yudho Bintoro, Pradana Zaky Romadhon, Satriyo Dwi Suryantoro, Rusdi Zakki Aminy, Choirina Windradi, Krisnina Nurul Widiyastuti

**Affiliations:** 1 Department of Internal Medicine, Airlangga University, Faculty of Medicine, Surabaya, East Java, 60131, Indonesia; 2 General Teaching Hospital Dr. Soetomo, Surabaya, East Java, 60286, Indonesia; 3 Universitas Airlangga Hospital, Surabaya, East Java, 60115, Indonesia

**Keywords:** priapism, chronic myeloid leukemia, cytoreduction, penile-aspiration, cancer

## Abstract

Priapism in chronic myeloid leukemia (CML) appears to be an infrequent manifestation as well as a crucial emergency. Here, we report an 18-year-old male presenting with a persistent erection of penis for 20 days. We evaluate and compare the reported cases during the past 20 years discussing the management of CML patients experiencing priapism. Cytoreductive therapy followed by leukapheresis, the administration of tyrosine kinase inhibitor, and intra-cavernosal blood aspiration may resolve the symptoms of priapism. Early intervention for cytoreduction and aspiration are the pivotal keys to successfully impeding the complications.

## Introduction

Priapism is a urological emergency due to persistence of an erection lasting more than 4 hours, whether or not it is related to sexual influence. ^1^ Priapism is a rare condition with an incidence of 1–5 cases per 100,000 people per year. Penile erection in priapism is regularly painless. There are two types of priapism, namely low-flow priapism and high-flow priapism. Low-flow priapism is provoked by a pathological condition of low venous blood flow causing stasis in the penile vessels. This condition is an emergency condition that can result in cell damage and fibrosis, so it often requires immediate therapy. Meanwhile, high-flow priapism is caused by increased blood flow to the sinusoid arteries without offsetting the flow to the veins. One of the causes of high-flow abnormalities is penile injury, while low-flow priapism is commonly caused by blood disorders such as sickle cell anemia and chronic myeloid leukemia (CML). ^2^^–^^4^

Hematological abnormalities account for 20% of the incidence of priapism while leukemia cases account for 1–5% of priapism in men. The theory related to the occurrence of priapism is the dysregulation of nitric oxide (NO) in penile vascularization. This occurs due to changes in NO synthase enzyme activity which cause a decrease in NO production by the corpora cavernosa. This ischemic condition induces platelet aggregation and thrombus and tissue damage. The hematologic condition generates priapism with decreased NO which interferes with smooth muscle tone controlling penile tumescence. Hyperviscosity conditions due to leukocytosis and adenosine-opiorphins abnormalities is also involved in this condition. ^1^

Currently, the approach to treating CML patients with priapism uses a modality of combination of systemic therapy (chemotherapy with hydroxyurea or tyrosine kinase inhibitors and leukapheresis) and local intracavernosal therapy. Some cases with late manifestations cause erectile dysfunction, gangrene and penile abscess. ^5^ This case report and review aims to discuss the clinical characteristics and outcomes of CML patients who experience complications in the form of priapism.

## Case

**Figure 1.**
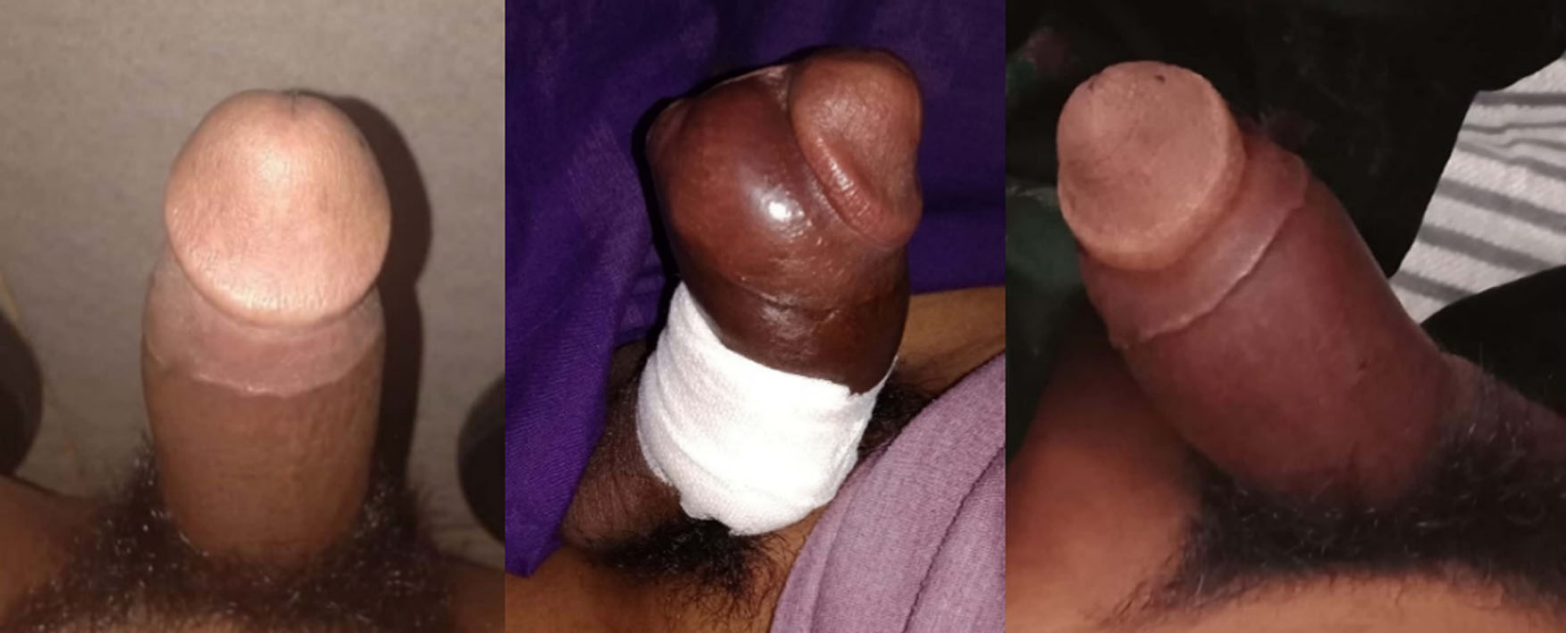
Penis at day 2, day 6 (after intracavernosal blood aspiration), and day 9.

An 18-year-old unmarried male student, presented at the ER complaining of persistent erection of the penis. The patient complained of persistent erected penis for 20 days before admission. There was no phase without an erection during those 20 days. Previously, there was no history of trauma to sexual stimulation, or consumption of certain drugs. The patient also complained of mild genital pain along with the onset of erection. There were no complaints about discoloration of the penis, becoming reddish, bluish, or pale. There was no sensation of numbness or numbness. The patient could urinate normally (see Figure 1).

The patient complained of alternating ringing in his right and left ears for 15 days accompanied by blurred vision. The patient also felt that his left side of stomach was slowly enlarging for 5 months. There was no bleeding and fever. Before coming to the ER, the patient was hospitalized at the regional hospital and received a blood transfusion and was diagnosed with a blood disorder.

On physical examination, there was no anemia and icterus. The spleen was palpable showing Schuffner 4 and Hackett 3. There was no enlargement of the lymph nodes. His laboratory findings were as follows: hemoglobin 10.4 g/dL; leucocytes 421,000 cells/mm ^3^; platelets 407,000 cells/mm ^3^; white blood cells differential 4.3/6.8/81.3/4.9/2.7; blood urea nitrogen 9 mg/dL; serum potassium 0.5 mg/dL, uric acid 6.5 mg/dL. Peripheral blood smear showed normochromic anemia, normocytic anisopoikilocytosis, leukocytosis (3% myeloblasts, 6% promyelocytes, 4% myelocytes, 2% metamyelocytes, 5% stab neutrophils, 63% segment neutrophils, 4% eosinophils, 6% basophils, 5% lymphocytes, 2% monocytes, atypical lymphocytes (+)) concluded as CML. The patient received hydroxyurea 2000 mg once daily at night, paracetamol 500 mg TID, and an urgent leukapheresis.

The patient underwent leukapheresis once per day (a total of three times since initial admission) with gradual improvement. Unfortunately, on the fourth day of treatment the patient felt a penis erection again with pain on a scale of 0–5. Local examination of the genitalia showed a maximal erected penis, with no discoloration indicative of hyperemia, cyanosis, or pallor. Blood gas analysis showed pH 6.95, pCO _2_ 64 mmHg, HCO _3_ 14 mEQ/L, Be -18 unit, so it was concluded that the patient had ischemic priapism. Therefore, the patient underwent urologic intervention by intracavernous aspiration producing 150 mL blood. Not long after the procedure, the patient's penis returned to an erection with bleeding from the puncture wound. It was decided that the patient undergo leukapheresis.

On the eighth day of treatment, the erection improved and the patient reported 1 on a pain scale. Quantitative *BCR–ABL* examination showed a positive result of 65% so that the administration of hydroxyurea was stopped and replaced by imatinib 400 mg once daily at night. On the twelfth day of treatment, the erection was completely resolved and the patient was successfully discharged from the hospital.

## Discussion

This review presents data on patients who have priapism due to CML (see Table 1). The results of this review indicate that cases of priapism occurred in the age range 9–53 years and the average patient had episodes of priapism for 18 h to 7 days. Not all patients with priapism showed a typical clinical examination of CML in the form of splenomegaly but all of these patients had a hyperleukocytosis profile with a leukocyte count >200,000 cells/mm ^3^. Some of them are equipped with data peripheral blood smear with excessive blast and identification of *BCR–ABL* gene. Only one case reported by Minckler *et al.* who reported a resolved erection with a cold shower but most cases needed medical and intervention therapy. ^6^ Although the duration of symptoms varied, four cases reported complications following an episode of priapism. Patients with unfavorable outcomes received hydroxyurea, imatinib but failed to undergo urological emergency therapy in the form of failure of intra-cavernosa aspiration, surgical intervention and embolization.

**Table 1.** Case report review from last than twenty years.

No	Author	Country	Year	Age	Duration of priapism	Diagnosis of CML	Treatment of CML	Treatment of priapism	Outcome of the treatment
1	Gaye *et al*. ^4^	Senegal	2020	46	48 hours	White Blood Cell: 526000/mm ^3^, Platelets: 412000/mm ^3^, Myelogram result: bone marrow hyperplasia. Karyotyping: Translocation between chromosomes 9 and 22	Imatinib (the dosage wasn’t mentioned)	Aspiration of corpora cavernosa, injection of phenylephrine, hydrocycarbamide	Success
			2020	9	36 hours	White Blood Cell: 82000/mm ^3^, Platelets: 81000/mm ^3^, BMA: acute myeloid leukemia	Vincristine and Prednisolone	penile skin refrigeration, rehydration, puncture of corpora cavernosa, injection of phenylephrine	Success
2	Rajabto *et al*. ^9^	Indonesia	2020	44	4 days	physical exam: pale skin, conjunctival pallor, leukemic retinopathy in both eyes. Schuffer 2.	IV fluid, Allopurinol 300 mg, Sodium bicarbonate 500 mg 3 times daily, hydrocyurea 1 gram three, Imatinib 400 mg times a day	aspiration of penile corpus, injection of epinephrine	suffered ED
						Labs: anemia, hyperleukocytosis, microcytic hypochromic, anisopoikilocytosis, fragmentocytes, polychromic erythrocytes, a left shift, platelet count (355,000/μL), and hyperleukocytosis (399.560/μL).			
						Positive BCR-ABL1			
						BMA: hypercellularity			
3	Dhar *et al*. ^11^	India	2019	52	4 hours	Physical examination: massive splenomegaly of 8 cm below the left costal margin along with hepatomegaly of 3 cm below right costal margin.	Hydroxyurea 500 mg TDS, Imatinib OD, Allupurinol 300 mg OD, adequate hydration	needle aspiration --> didn’t work, went for Winters procedure	Success
						Blood count: left sided granulopoeis, total leucocyte count of 239×109/L and platelet count of 625×109/L.			
						BMA: findings of CML			
						positive translocation of BCR-ABL			
4	Becerra *et al*. ^12^	Mexico	2018	52	6 day evolution	WBC: 282.000, platelets: 368×10 ^3^/mm ^3^	dastinib 100 mg/day+G15	corpora cavernosa irrigation and surgery penis shunts	Success
						BMA: acute phased CML			
						translocation t (9:22)(q34;q11.2) with P210 BCR-ABL1 fusion transcriber			
5	Khan *et al*. ^13^	Pakistan	2018	16	264 hours	Leukocyte count: 614.8×10 ^9^, platelets 709×10 ^12^/L, peripheral smear: myeloid hyperplasia, neutrophilia. BMA: myeloid hyperplasia. Detection of BCR-ABL	Hydroxyurea, allopurinol	Glans-cavernosal shunt	Achieved detumescence, No info on ED
6	Qu *et al*. ^14^	China	2018	18	72 hours	Hepatosplenomegaly 2-3 cm under arcus costae, blood count: white blood cell (WBC) 257×10 ^9^/L and platelets (PLT) 5450×10 ^9^/L	Imatinib	Caverosa-corpus spongiosum shunt	No ED at 3 months follow up
7	Clark *et al*. ^15^	USA	2018	13	3 days	Blood count: WBC count of 350,000/mL (350×10 ^9^/L) and platelet count of 450×10 ^3^/mL (450×10 ^9^/L). Flow cytometry of blood: granulocytosis with no increase in blasts	leukapharesis, IV fluids, hydroxyurea, allupurinol, Imatinib	phenylephrine injection, three times corporeal irrigation	improved with phallus rigidity and tenderness
						BMA: Philadephia chromosome			
8	Kumar *et al*. ^16^	India	2018	47	5 days	Hepatosplenomegaly, WBC: 279×109, 91.2%BCR	Hydroxyurea, Imatinib	Aspiration and irrigation with phenlyephrine, Winter's T Shunt	Successful treatment
				42	7 days	Splenomegaly 6 cm below costal margin, WBC: 390×109/L, 70,7% BCR-ABL ratio	Hydroxyurea, Imatinib	Aspiration and irrigation	Successful treatment
				28	6 days	No hepatosplenomegaly, WBC: 206×109/L, 75.3% BCR-ABL ratio	Hydroxyurea, Imatinib	Aspiration and irrigation with phenlyephrine, Winter's T Shunt	Successful treatment
9	Sun *et al*. ^5^	USA	2018	27	8 years, persistent erection 9 hours	Labs: anemia, WBC 450,010, Platelets 509,000/mm ^3^ BMA: 2% blasts, hypercellular bone marrow, granulocytic hyperplasia, small megakaryocytes. BCR-ABL did not reveal clonal evolution.	Leukapheresis, hydoxyurea 500 mg daily, allopurinol 300 mg daily, Imatinib 400 mg daily,	Corporal bpody aspiration, 1 dose of phenylephrine injection	Successful treatment
10	Huei *et al*. ^17^	Malaysia	2018	28	48 hours	hepatomegaly 2cm below right costal margin, splenomegaly, anemia, WBC 294×10 ^9^, platelets: 94×10 ^9^/L Peripheral blooad smear: hyperleucocytosis, blast cells	Hydroxyurea, allupurinol, intravenous Cytarabine	Intracavernosal aspiration, phenylephrine irrigation--> detumescent --> reccurent erection --> corpoglandular shunt	Successful treatment
11	Minckler *et al*. ^6^	USA	2017	18	3 month intermittent	WBC: 588×10 ^3^/uL, platelets: 109×10 ^3^/uL	Hydroxyurea transtition to imatinib 400 mg daily	Penile irigation and aspiration	Success
						peripheral blood: hyperleukocytosis with absolute neutrophilia and a peripheral blast count of 2%.			
						bone marrow aspirate and biopsy: hypercellular marrow with 4% blasts			
						FISH analysis: translocation t(9:22)			
12	Nerli RB *et al*. ^7^	India	2016	19	duration: 24 hours	WBC 296800, platelet 936,000/mm ^3^, BMA: hypercellular, increased megakaryocytes	Hydroxyurea 1.5 gram daily, Imatinib 40 mg daily Allupurinol 300mg daily per oral	Irrigation, decompression	Successful
13	Ergenc H *et al*. ^18^	Turkey	2015	18	duration: 72 hours	Hepatosplenomegaly 2-3 cm under arcus costae, anemia, WBC 100.000, platelets 1,002,000/mm, peripheral blood smear: immature leukocytes. BMA: hypercellularity with myeloid hyperplasia, positive BCR-ABL translocation	Imanitib 400 mg once daily, allopurinol 300 mg once daily, leukapharesis	not mentioned	Success
14	Shaeer *et al*. ^2^	Egypt	2015	21	6 days	palpable splenomegaly, WBC 410000, Philadelphia chromosome translocation	Leukapharesis, Imatinib 400 mg daily	failed several cavernosal aspiration and injection of epinephrine --> penile prosthesis	No complication throughout 6 months-follow up
15	Osorio *et al*. ^19^	Spain	2014	24	14 hours, the second episode. The first episode was 4 months ago	WBC: 177.15×109, platelet was not mentioned, cytogenic diagnosis: showing CML	Imatinib	Corpora cavernosa aspiration, intracavernosa fenilefrin injection	not mentioned
				29	6 hours, the second episode. The first episode was less than a month ago	WBC: 402.24×109, platelet was not mentioned positive BCR-ABL	hyrdoxyurea	Corpora cavernosa aspiration, intracavernosa fenilefrin injection	not mentioned
16	Hazra *et al*. ^20^	India	2013	14	24 hours	Splenomegaly 6 cm below the left costal margin, anemia, WBC 226900, platelets 310,000/uL, Peripheral blood smear: immature leukocytes in various stages. BMA: CML.	Hydroxyurea 50 mg/kgBB/day, Allupurinol 300 mg/day	Cavernosal aspiration and phenylephrine irrigation	No recurrence at 2-months-follow-up
17	Veljkovic *et al*. ^21^	Serbia	2012	16	24 hours	Splenomegaly 4 cm below costal margin, WBC 320×109/L, Platelet (Plt) 417×10 ^9^/L BMA: extreme hypercellularity, BCR/ABL positive	leukapharesis, cytoreductive chemotherapy	leukapharesis	no follow up
18	Paladino *et al*. ^3^	Spain	2011	16	48 hours	Splenomegaly, WBC 312.000, PLT: 60.000/mm ^3^ BMA: showing CML	no mention	Corpora cavernosa drainage	Erectile dysfunction
19	Gupta *et al*. ^22^	India	2009	12	48 hours	Hepatosplenomegaly below the costal margins, anemia, WBC: 346×109/L, platelet count of 40,000/mm ^3^, peripheral blood smear: immature myeloid leukocytosis. Cytogenesis: philadelphia chromosome. BCR-ABL transcript was positive	hydroxyurea 4g/day IV fluid 3L/day, allopurinol, Imatinib 400mg/day, leukapharesis	Terbutaline 0.125 mg subcutaneously	Resolved by 24 h
20	Ilais Tazi ^23^	Morocco	2009	33	duration: 22 hours	Palpable splenomegaly 4 cm below left costal margin, WBC: 400000/mm ^3^, platelets 1200000/mm ^3^. Peripheral blood smear: immature leukocytes. Karyotyple analysis: Ph1 chromosome, myeloid hyperplasian in the bone marrow.	Imatinib	Aspiration	Success
21	Castagnetti *et al*. ^24^	Netherland	2008	9	several days	splenomegaly, anemia, WBC: 509×109/L, philadelphia chromosome, BCR-ABL +	Hydroxyurea 1.5mg/m ^2^/day, Cyclophosphamide 250 mg/m ^2^/day for 2 days, leukopharesis	cytoreduction, antibiotics, anticoagulants	Fully resolved after 1 month
				9	96 hours	mild splenomegaly	Hydroxyurea 1g/m ^2^/day	LMWH 90 units/kg SQ BID for 1 month, metamizole	fully resolved after 3 months
				9	9 hours	hepatosplenomegaly	Cyclophosphamide 250 mg/m ^2^/day for 2 days, leukapharesis	LMWH 90 units/kgBB SQ BID for 9 days, metamizole, morphine	fully resolved after 20 days
22	Yoshida *et al*. ^25^	Japan	2007	29	48 hours	WBC 263000	Imatinib mesylate	Winter procedure	no evidence of recurrent
23	Lopez *et al*. ^26^	Spain	2004	29	10 hours	WBC 414×10 ^9^/L, BMA: hypercellularity, PLT: 1100 × 10 ^9^/L	corpora cavernosa aspiration, phenylephrine injection	corpus cavernosum aspiration, fenilefrin injection	Successful treatment
24	Ponniah *et al*. ^27^	United Kingdom	2004	19	18 hours	WBC 513×109/L	Leukapharesis	failed cavernosal aspiration + leukapharesis	No ED on follow up
25	Dogra *et al*. ^28^	India	2003	18	10 days	hepatosplenomegaly, anaemic, WBC 320000, PLT was not mentioned	Intravenous hydration, furosemide, sodium bicarbonate, hydroxyurea, allopurinol, leukapharesis	Winters Procedure	impotent and enlarged penis at 3-months follow up
26	Meng-Wei Chang *et al*. ^8^	Taipei	2003	21	19 hours	Hepatomegaly 6 cm below right arcus costae, Splenomegaly 7 cm below left arcus costae, anemia, WBC 216800, Platelet 1746,000/mm ^3^	Interferon alfa-2a (6MIU/vial), allopurinol 300 mg daily	Aspiration, epinephrine irigation	Success
27	Guerra *et al*. ^29^	Spain	2002	53	12 hours	WbC 968×109/L	Hydroxyurea	Corpora cavernosa aspiration	Successful treatment
28	Murayama *et al*. ^30^	Japan	2001	14	4 days	WBC 510000, BMA: myeloid hyperplasia, karyotype analysis: chromosome Ph1	urokinase, hydroxyurea	embolization of bilateral pudendal artery	Reduced sexual potency
29	Rojas *et al*. ^31^	Chilli	1998	22	duration: 36 hours	none	Leukapharesis	Surgical intervention	Unsuccessful (post treatment sexual dysfunction)

In this case, the patient was 18 years old. Based on the literature, patients in every age group are at risk of developing priapism. However, there are two peaks in the age distribution that tend to experience this condition. The peak in pediatric age is between 5 and 10 years, especially in patients with sickle cell disease. While the second peak age is at sexually active phase between 20 and 50 years. Apart from being a condition of hypercoagulability, this condition may also be related to the abuse of erectile drugs. ^7^

History and physical examination are important when encountering cases of priapism. Laboratory tests are required to check for impaired coagulation and serum electrolytes. Some patients who are at high risk for priapism include users of intracorporal injection therapy for erectile dysfunction, coagulation disorders such as sickle cell disease and CML. ^2^^,^^4^ In CML, hyperleukocytosis is thought to be the main cause of priapism. The main mechanism is the aggregation of leukemia cells in the corpora cavernosa and dorsal veins of the penis. Another thing that underlies the mechanical pressure in the abdominal veins due to the enlargement of the spleen. ^1^

The data needed in the management of patients with this case are erection duration, pain scale, trauma, complete blood count, peripheral blood smear, penile blood gas analysis, bone marrow and polymerase chain reaction for *BCR–ABL1* if necessary. ^1^^,^^2^^,^^4^ In CML, the most common type of priapism is the ischemia one (veno-occlusive). Patients usually complain of rigid erection, which may be accompanied by pain characterized by reduced to no cavernous blood flow at all. Priapism that lasts for more than 4 hours indicates a compartment syndrome and may require emergency medical intervention. ^8^

The American Urological Association recommends that systemic treatment of an underlying disorder, like CML, should not be the only one therapy for ischemic priapism. In this case, the patient has had an erectile episode since 20 days who most likely has had a compartment syndrome so that the intra-cavernous aspiration is required. ^1^

The intra-cavernous aspiration procedure can be accomplished by giving the anesthetic injection first under the symphysis pubis. The penis is tied with a tourniquet followed by insertion of a 16–18-Gauge bivalve intravenous catheter into the corpus cavernosum. When the two corpora are fused, aspiration of 20–30 mL of blood can be undertaken. This procedure has 30% chances of success. ^8^^,^^9^

Systemic therapy is often used to reduce hyperviscosity is cytoreductive therapy such as high-dose hydroxycarbamide and tyrosine kinase inhibitors (TKI) with or without apheresis procedures. The dose of hydroxycarbamide that can be given is 2–6 grams divided into four doses per day that can reduce leukocytes by almost 60% in 24–48 h. In addition, TKI administration such as imatinib can be administered as soon as the diagnosis is confirmed. The recommended dose of imatinib is 400 mg once daily in the chronic phase, 600–800 mg once daily in the accelerated phase and 800 mg once daily in a blast crisis. ^9^ In the case of CML in general, The IRIS study describes the effectiveness of imatinib therapy for complete hematological response (CHR), major cytogenetic response (McyR) and complete cytogenetic response (CcyR). ^4^

Leukapheresis can cause a rapid decrease in intravascular leukemia cells and improve tissue perfusion as well as complaints related to leukostasis (generally show pulmonary and central nervous system manifestations). One leukapheresis procedure can reduce the leukocyte count by 30–60%. Albeit leukapheresis can reduce leukocytes significantly and rapidly compared to chemotherapy, several studies have shown high all-cause mortality. According to 2016 apheresis guidelines, grade 1B of acute myeloid leukemia is recommended (strong recommendation, moderate quality evidence) with category 2 (second-line therapy), while for acute lymphoblastic leukemia cases grade 2C is recommended (weak recommendation, low quality evidence) with category 3 (the role of apheresis is not very clear). In this guideline, leukapheresis recommendations are not stated in cases of chronic myeloid leukemia. ^10^ Several cases of priapism in this case review reported a successful combination of leukapheresis therapy with systemic oral CML therapy. Only one case by Rojas *et al.* underwent leukapheresis but failed to improve.

**Figure 2.**
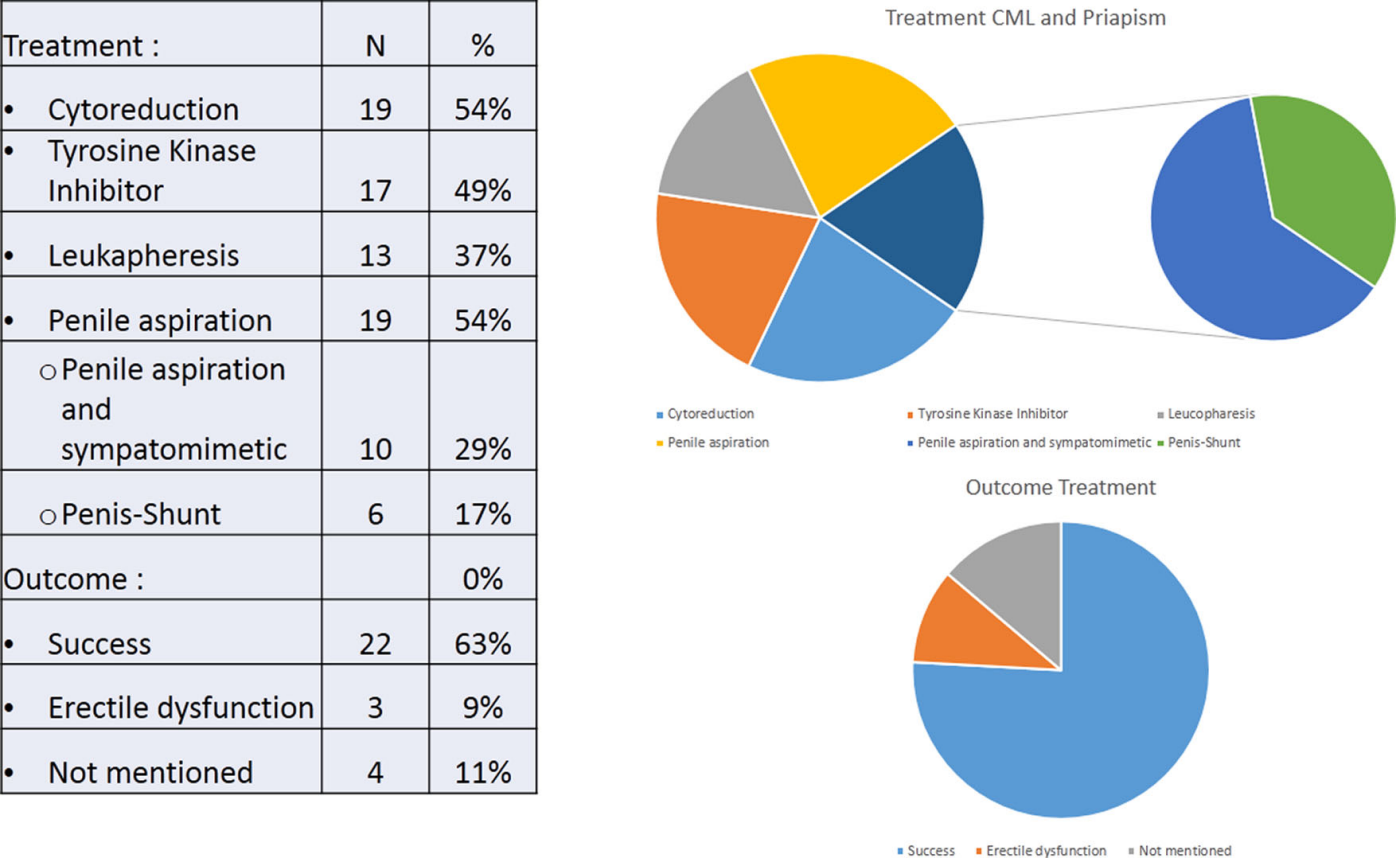
Treatment and outcome from priapism and CML.

This case report and review presents a comparative presentation of patient characteristics, clinical characteristics of CML, laboratory profile, and therapeutic intervention for CML with priapism. Clinical presentation and early intervention are the keys to successful therapy in preventing complications. Systemic intervention combined with intraurethral therapy increase the success rate (see Figure 2).

## Consent

Written informed consent for publication of their clinical details and/or clinical images was obtained from the patient.

## Data availability

All data underlying the results are available as part of the article and no additional source data are required.
