# Supplementary material for: Case Report: Priapism as The Clinical Presenting Feature of Chronic Myeloid Leukemia: Case Report and 20-Year Literature Review
Source: F1000Res. 2022 Jan 13;10:571. Originally published 2021 Jul 15. [Version 2] doi: 10.12688/f1000research.53365.2 (PMC8762679; doi:10.12688/f1000research.53365.2)
Supplement: Peer review history

# Peer review: Reviewer response for version 2

**DOI:** 10.5256/f1000research.80090.r119664

**Published:** 2022-01-14

**Authors:** Ritu Gupta

**Affiliations:** 1 Laboratory Oncology Unit, Dr B.R. Ambedkar IRCH, All India Institute of Medical Sciences (AIIMS), New Delhi, New Delhi, Delhi, India

The authors have summarized the subject adequately and I have no further comments.

Are enough details provided of any physical examination and diagnostic tests, treatment given and outcomes?

Yes

Is the case presented with sufficient detail to be useful for other practitioners?

Yes

Is sufficient discussion included of the importance of the findings and their relevance to future understanding of disease processes, diagnosis or treatment?

Partly

Is the background of the case’s history and progression described in sufficient detail?

Yes

Reviewer Expertise:

Hemato-Oncology, Genomics, Single-cell sequencing, Flow cytometry

I confirm that I have read this submission and believe that I have an appropriate level of expertise to confirm that it is of an acceptable scientific standard.

# Peer review: Reviewer response for version 1

**DOI:** 10.5256/f1000research.56738.r100511

**Published:** 2021-12-02

**Authors:** Ritu Gupta

**Affiliations:** 1 Laboratory Oncology Unit, Dr B.R. Ambedkar IRCH, All India Institute of Medical Sciences (AIIMS), New Delhi, New Delhi, Delhi, India

Priapism is an unusual complication of hematological malignancy with hyperleukocytosis and may be the presenting feature, especially in chronic leukemia as observed in this case. 

 The authors have described the clinical features, investigations, and management of the index case and reviewed the literature on the association of priapism with CML.

 I have a few comments/suggestions on this manuscript as detailed below:

1. The title is too long and can be abbreviated.
2. Review of hematological malignancies presenting as priapism i.e. including CLL and Acute leukemia would benefit the readers in developing insight on the conditions in which priapism could be the presenting feature. A few references include the following: Johnson *et al*. (2020 ^1^), Ali *et al. *(2021 ^2^) and Gogia *et al*. (2012 ^3^).
3. The meaning of some of the sentences is not clear in several places. The authors may focus on improving the language of the paper.

Are enough details provided of any physical examination and diagnostic tests, treatment given and outcomes?

Yes

Is the case presented with sufficient detail to be useful for other practitioners?

Yes

Is sufficient discussion included of the importance of the findings and their relevance to future understanding of disease processes, diagnosis or treatment?

Partly

Is the background of the case’s history and progression described in sufficient detail?

Yes

Reviewer Expertise:

Hemato-Oncology, Genomics, Single-cell sequencing, Flow cytometry

I confirm that I have read this submission and believe that I have an appropriate level of expertise to confirm that it is of an acceptable scientific standard, however I have significant reservations, as outlined above.

## References

[1] : Which patients with ischaemic priapism require further investigation for malignancy?. *Int J Impot Res* .2020;32(2) : 10.1038/s41443-019-0141-z 195-200 10.1038/s41443-019-0141-z 30996267

[2] : Priapism in lymphoproliferative disorders: A systematic review. *Hematol Oncol Stem Cell Ther* .2021; 10.1016/j.hemonc.2021.05.003 10.1016/j.hemonc.2021.05.003 34157311

[3] : Priapism as an initial presentation of chronic lymphocytic leukemia. *Leuk Lymphoma* .2012;53(8) : 10.3109/10428194.2012.656636 1638-9 10.3109/10428194.2012.656636 22242818

# Response

**Published:** 2021-12-03

**Authors:** Pradana Zaky Romadhon

**Affiliations:** Airlangga University, Indonesia

Firstly, thank you for the detailed review and advice. We have simplified the title and removed unclear sentences then replaced them with more understandable ones.

# Response

**Published:** 2021-12-04

**Authors:** Pradana Zaky Romadhon

**Affiliations:** Airlangga University, Indonesia

Dear Ritu Gupta, 

 We already just submitted our new version of the manuscript. We also have included one of the references recommended by you in our discussion. Hope it will upgrade our manuscript quality. Thanks again.

# Peer review: Reviewer response for version 1

**DOI:** 10.5256/f1000research.56738.r89693

**Published:** 2021-10-18

**Authors:** Wulyo Rajabto

**Affiliations:** 1 Division of Hematology-Medical Oncology, Department of Internal Medicine, Dr. Cipto Mangunkusumo General Hospital, Faculty of Medicine, University of Indonesia, Jakarta, Indonesia

This case report emphasizes the importance of priapism as the rare clinical presentation of chronic myeloid leukemia so that as a clinician we should think if there is patient with priapism the secondary causal is chronic myeloid leukemia. The treatment of priapism consists of: 1) Local factor by urologist who performs intra cavernous aspiration 2) The systemic factor by hematologist who administers leucapheresis (mechanical and drug eg. Hydroxyurea) and TKIs such as Imatinib.   

 I find the title of this manuscript indeed captivating. Besides describing the priapismus phenomenon in CML, the author also showed to us a comparison study among several previously published cases known worldwide, that I think it is a very interesting plus point.

- Title: I believe it is very interesting, straightforwardly describes the case.
- Introduction: I believe it contains concise reasoning why the author brought up this case, emphasizes the rare of similar cases, and interestingly presents one of CML emergencies.
- Case presentation: The author successfully managed to present the case elaborately along with valid data.
- Discussion: The author describes the case comprehensively, referred to similar case studies before, from the clinical course to the outcome, as mention on table 1.

Are enough details provided of any physical examination and diagnostic tests, treatment given and outcomes?

Yes

Is the case presented with sufficient detail to be useful for other practitioners?

Yes

Is sufficient discussion included of the importance of the findings and their relevance to future understanding of disease processes, diagnosis or treatment?

Yes

Is the background of the case’s history and progression described in sufficient detail?

Yes

Reviewer Expertise:

CML, lymphoma, anemia

I confirm that I have read this submission and believe that I have an appropriate level of expertise to confirm that it is of an acceptable scientific standard.
